# Optimizing an immunomodulatory potency assay for Mesenchymal Stromal Cell

**DOI:** 10.3389/fimmu.2022.1085312

**Published:** 2022-12-12

**Authors:** Stine Bangsgaard Hansen, Lisbeth Drozd Højgaard, Jens Kastrup, Annette Ekblond, Bjarke Follin, Morten Juhl

**Affiliations:** ^1^ Cell2Cure, Cardiology Stem Cell Centre, The Heart Centre, University Hospital Rigshospitalet, Copenhagen, Denmark; ^2^ Cell2Cure, Birkerød, Denmark

**Keywords:** mesenchymal stromal cell, adipose tissue-derived stromal cell, lymphocyte proliferation assay, mitogen titration, flow cytometry, functional assay, potency assay development

## Abstract

The expeditious progress of Mesenchymal Stromal Cells (MSC) for therapeutic intervention calls for means to compare differences in potency of cell products. The differences may be attributed to innumerable sources including tissue origin, production methods, or even between batches. While the immunomodulatory potential of MSC is recognized and well-documented by an expansive body of evidence, the methodologies and findings vary markedly. In this study, we utilized flowcytometric analysis of lymphocyte proliferation based on cryopreserved peripheral blood mononuclear cells for quantification of the inhibitory effect of MSC. Technical aspects of fluorescent staining and cryopreservation of peripheral blood mononuclear cells were evaluated to obtain optimal results and increase feasibility. A range of common specific and unspecific mitogens was titrated to identify the conditions, in which the effects of Adipose tissue-derived Stromal Cells (ASC; a type of MSC) were most pronounced. Specific stimulation by antibody-mediated activation of CD3 and CD28 *via* TransAct and Dynabeads lead to substantial proliferation of lymphocytes, which was inhibited by ASC. These results were closely mirrored when applying unspecific stimulation in form of phytohemagglutinin (PHA), but not concanavalin A or pokeweed mitogen. The mixed lymphocyte reaction is a common assay which exploits alloreactivity between donors. While arguably more physiologic, the output of the assay often varies substantially, and the extent of proliferation is limited since the frequency of alloreactive cells is low, as opposed to the mitogens. To heighten the proliferative response and robustness, combinations of 2-5 donors were tested. Maximum proliferation was observed when combining 4 or more donors, which was efficiently suppressed by ASC. Several desirable and unfavorable traits can be attributed to the tested stimuli in the form of keywords. The importance of these traits should be scored on a laboratory-level to identify the ideal mitogen. In our case the ranking listed PHA as the most suited candidate. Developing robust assays is no trivial feat. By disclosing the full methodological framework in the present study, we hope to aid others in establishing functional metrics on the road to potency assays.

## Introduction

Multipotent mesenchymal stromal cells (MSC) were first described decades ago (1–3) and the interest in using the cells for therapy has only been increasingly since. This is attributed to their favorable safety profile and multiple beneficial properties (4–6). Among these properties, MSC have immunomodulatory effects on different immune cells of both the innate and the adaptive immune system (7–10). MSC can be isolated from practically any vascularized tissue, including bone marrow, umbilical cords, and adipose tissue (1, 11–13). At Cardiology Stem Cell Centre, we have developed methods for isolation and expansion of MSC derived from adipose tissue (Adipose tissue-derived Stromal Cells, ASC) as a therapeutic off-the-shelf allogeneic cell product in compliance with Good Manufacturing Practice (5, 12, 14)

Despite more than 75,000 publications on MSC (15) and clinical trials showing promising results, few companies have succeeded to commercialize MSC as a cell-based therapeutic product (13, 16). MSC arise from and constitutes a heterogeneous cell population and the functional properties vary from donor to donor (17) making each batch of allogenic MSC unique (18). Therefore, it is required that the cell product is tested for quality control during the manufacturing process and before release for clinical use. These tests include purity, impurities, identity, sterility, cell viability, and potency (19, 20), where the development of an appropriate potency assay often is the most challenging part (18, 21).

Numerous *in vitro* assays have been put forth to evaluate the immunomodulatory properties of MSC (17, 18, 22, 23). For an immune potency assay, appropriate responder cell(s) must be chosen to evaluate the expected mode of action exerted by MSC. For lymphocyte proliferation assays, a heterogeneous cell population like peripheral blood mononuclear cells (PBMC) consisting of lymphocytes, monocytes, and dendritic cells (24, 25) or isolated responders such as T cells (23) can be used. Lymphocytes need to be activated to become effector cells (25). Different stimuli have been discovered to induce lymphocyte proliferation including unspecific mitogens like phytohaemagglutinin (PHA) (26) or specific antibody-mediated activation of CD3 associated with the T cell receptor and CD28 for co-stimulatory signaling (27). Alternatively, lymphocyte proliferation can be induced by donor differences to elicit an alloreactive response, the mixed lymphocyte reaction (MLR) (28).

In this study we aimed to investigate and optimize factors to obtain the most feasible and robust potency assay to quantify the suppressive effect of ASC on lymphocyte proliferation. These factors included induction of proliferation by stimulation with five different mitogens or by MLR, the impact of cryomedia, carboxyfluorescein succinimidyl ester (CFSE)-labelling prior to cryopreservation, and the use of monocyte-depleted PBMC.

## Materials and methods

### Peripheral blood mononuclear cells

Human PBMC were isolated from six buffy coats generated from citrate-phosphate-dextrose treated blood (Department of Clinical Immunology, Rigshospitalet, Denmark) donated by consenting, anonymized, healthy donors according to Danish Transfusion Medical Standards, on the same day blood was donated. The buffy coats were diluted to a volume of 180 ml in phosphate-buffered saline (PBS,Gibco) and layered on LymphoPrep density gradient medium (Alere Technologies) in LeucoSep tubes with an inert porous membrane (50 ml, Greiner Bio-One) and centrifuged at 800g for 15 minutes (room temperature; RT). Subsequent centrifugations were carried out at 300g for 5 minutes (RT). The supernatant was discarded, the interphase consisting of PBMC transferred to new centrifuge tubes and washed four times with PBS. Prior to the final centrifugation the PBMC were filtered through a 100 µm cell strainer. The cells were counted (Nucleocounter NC100, ChemoMetec) and 2×10^8^ viable cells of the PBMC suspension were put aside for experimental setups exploring formulation of cryomedium, monocyte depletion, and CFSE-labelling (described below). The remaining PBMC were resuspended in 90% fetal bovine serum (FBS,Gibco) supplemented with 10% dimethyl sulfoxide (DMSO, WAK – Chemie Medical GmbH), henceforth referred to as standard cryomedium, at a final cell concentration of 1×10^7^ cells/ml. The combination of FBS and DMSO has previously been optimized (29). 1 ml cell suspension was aliquoted into cryotubes (Nunc) and subsequently placed in CoolCell (Biocision). The CoolCell was cooled for 10 minutes at 4°C before transfer to -80°C overnight for controlled freezing (-1°C/min). The following day the cryotubes were transferred to liquid nitrogen for long term storage. These six PBMC donors were applied for all experimental setups in this study.

### Alternative formulations of cryomedia

Aside from standard cryomedium, two alternatives were tested to eliminate xenogeneic components: human serum albumin (HSA, Sigma-Aldrich), and CryoStor CS10 (Biolife Solutions). HSA was prepared at a 13.9% (w/v) concentration in RPMI 1640 (Sigma-Aldrich), sterile-filtered, aliquoted, and stored at -20°C until use. Upon thawing, DMSO was added to generate a cryomedium consisting of 12.5% HSA and 10% DMSO in RPMI 1640 based on literature (30–32). CS10 is a GMP compliant cryomedium containing 10% DMSO, which is supplied ready-to-use. The cells were cryopreserved rate-controlled in CoolCell containers as previously described.

### Monocyte depletion of PBMC

All centrifugations in this procedure were carried out at 300g for 10 minutes at RT. From the original PBMC, 1×10^8^ cells were transferred to a new 50 ml centrifuge tube, washed in PBS, centrifuged, and resuspended in cold (4°C) degassed Magnetic-activated Cell Sorting (MACS) buffer consisting of PBS supplemented with 2 mM EDTA (Gibco) and 0.5% FBS. CD14-negative PBMC were separated from monocytes (CD14+) by MACS according to the manufacturers instruction (Miltenyi Biotec). In brief, 200 µl CD14 magnetic microbeads were added to the cell suspension, incubated for 15 minutes at 4°C, washed in MACS buffer, and loaded on a LS column in a MidiMACS separator. The column was washed thrice with MACS buffer. The CD14 negative cells were washed in PBS, and 1×10^7^ viable monocyte-depleted PBMC were cryopreserved in standard cryomedium as previously described.

### CFSE-labelling of PBMC

PBMC were thawed in water bath (37°C) for 10 minutes and diluted 1:10 in complete pre-warmed medium, consisting of 10% FBS in RPMI-1640 with 100 units/ml penicillin and 100 µg/ml streptomycin (Gibco). The cells were incubated 1 hour (37°C, 5% CO_2_) for recovery followed by centrifugation at 500g for 5 minutes at RT and labelled with 5 µM CFSE (BD Biosciences) in 2 ml PBS supplemented with of 2.5% FBS. For CFSE titration, see **Supplementary Materials Figure 1**. In agreement with Quah et al. (33), we have experienced that addition of FBS during staining reduces toxicity substantially and allows for longer incubations, increasing reproducibility by reducing inter-assay and operator variation. The cells were vortexed briefly and incubated in the water bath for 10 minutes (37°C) followed by three washes with 5% FBS in PBS. The cells were resuspended in complete medium and counted. 1×10^5^ CFSE-labelled PBMC were seeded per well in a 96-well plate (round bottom, tissue culture treated, Corning) in absence or presence of 5 µg/ml PHA-L (Sigma-Aldrich) in a final volume of 200 µl.

To determine ideal CFSE-labelling of PBMC prior to cryopreservation, staining was performed as described above at three different CFSE concentrations: 2.5, 5, and 10 µM. The cells were washed once in 5% FBS in PBS followed by resuspension in complete medium and allowed to recover in incubator (37°C, 5% CO_2_). The cells were cryopreserved in standard cryomedium as previously described.

### Adipose tissue-derived stromal cells

ASC were manufactured as a therapeutic cell product intended for clinical use as previously described (5, 12, 14). In brief, liposuction was performed on healthy consenting donors to obtain lipoaspirate from which the stromal vascular fraction (SVF) was isolated. The use of lipoaspirate has been approved by the Regional Research Ethics Committee (Region Hovedstaden), Denmark. Cardiology Stem Cell Centre holds an Authorization of tissue establishment for the handling of human tissues and cells licensed by The Danish Patient Safety Authority and a Manufacturing and Importation Authorization issued by the Danish Medicines Agency. The SVF was loaded into a bioreactor, Quantum Cell Expansion System (Terumo BCT), with medium consisting of Minimum Essential Medium alpha (α-MEM, Gibco), 100 U/ml penicillin, 100 µg/ml streptomycin (both Gibco) and 5% human platelet lysate (hPL, Sexton Biotechnologies). After expansion the cells were harvested with TrypLE (Gibco) and cryopreserved in CS10.

For this study, the therapeutic cell product was expanded for 2 additional passages. The cells were thawed in water bath (37°) for 5 minutes and cultured in a culture treated T175 flask (Nunc) with α-MEM supplemented with 5% hPL and 100 U/ml penicillin and 100 µg/ml streptomycin. The medium was changed twice a week. For passaging and harvesting the culture medium was discarded and the flasks were washed with PBS prior to addition of 1x TrypLE (Gibco). The cells were incubated in the incubator with TrypLE for 5-10 minutes until the cells had detached the surface and the cells were collected in a 50 ml centrifuge tube (Falcon) followed by centrifugation at 300g for 5 minutes (RT). Following 2 passages, ASC were cryopreserved in CS10 at a concentration of 1×10^6^ ASC/ml.

Prior to use, one vial of each donor was thawed in water bath (37°, 5 min) and diluted in complete medium followed by centrifugation. The cells were resuspended in complete medium and counted to generate a pool of 5 ASC donors, where each donor was equally represented, at final concentration of 1×10^5^ cells/ml. See **Supplementary Materials Figure 2** for comparison of single and pooled ASC. The ASC were seeded one day prior to co-culture with PBMC in a 96-well plate (round bottom, tissue culture treated), at a concentration of 2×10^4^ cells/well corresponding to 1:5 (ASC : PBMC).

### Lymphocyte proliferation assays

PBMC were thawed in water bath (37°C, 10 min) and diluted 1:10 in complete pre-warmed medium. The cells were incubated for 1 hour (37°C, 5% CO_2_) for recovery followed by centrifugation at 500g for 5 minutes at RT and labelled with CFSE.

To compare the proliferative response, PBMC were stimulated with PHA-L, concanavalin A (ConA), pokeweed mitogen (PWM) (Sigma-Aldrich), TransAct (Miltenyi Biotec), or Dynabeads (Gibco). Prior to use the Dynabeads were washed in Dynabuffer (0.1% bovine serum albumin (Sigma-Aldrich) and 0.5 M EDTA in PBS) and placed in a magnetic rack (Magnetic Particle Concentrator (MPC-6), ThermoFisher) for 1 minute. The Dynabuffer was discarded, and the beads were resuspended in complete medium to generate a concentration of 5 beads/cell which were further diluted in a 10-fold serial dilution (ranging from 5 beads/ml to 5×10^-4^ beads/ml). Lyophilized PHA-L, ConA, and PWM were reconstituted in PBS and stored at -20°C until use. Mitogens were diluted in complete medium to generate concentrations from 25 to 4×10^-2^ µg/ml in a 5-fold serial dilution. TransAct is a ready-to-use solution and was diluted in complete medium to generate concentrations from 1:20 to 1:2×10^5^ titer in a 10-fold serial dilution. Each CFSE-labeled PBMC donor was stimulated with the mitogens in absence or presence of ASC in triplicates and the assay elapsed for 5 days.

For MLR, the six PBMC donors were CFSE-labelled simultaneously. The cells were resuspended in complete medium and counted. Each donor was seeded in a pool of 2, 3, 4, or 5 PBMC donors leading to a combination of 15, 20, 15 and 6 unique lymphocyte reactions, respectively. The number of cells seeded from each donor depends on the number of pooled donors, however, the combined cell number was 1×10^5^ CFSE-labelled PBMC in 200 µl in absence or presence of ASC. The cells were seeded in quadruplicates, except from the experiment with the pool of 5 PBMC donors, which were seeded in octoplicates, and incubated for 8 days (37°C, 5% CO_2_). An 8-day time course were selected based on optimization (**Supplementary Material Figure 3**). The medium was partly changed 4 days after seeding by removing 100 µl supernatant and replacing with an equal amount of prewarmed complete medium.

The PBMC cryopreserved in HSA and CS10 and the monocyte-depleted PBMC, were thawed and labelled with 5 µM CFSE, and seeded alongside PBMC labelled with CFSE prior to cryopreservation in quadruplicates in 96-well plates, as previously described. The cells were cultured with complete medium in presence or absence of 5 µg/ml PHA-L and incubated for five days. The standard PBMC was included as control.

### Flow cytometry

5 or 8 days after the PBMC were seeded for mitogen and MLR assay, respectively, the assays were terminated. The cells suspension of PBMC stimulated with Dynabeads were harvested and transferred into 15 ml centrifuge tubes and subsequently placed in the MPC-6 for 2 minutes. The supernatant was collected, and diluted in PBS followed by centrifugation at 500g for 5 minutes (RT). The cells were resuspended in PBS and held in non-treated polypropylene 96-well plate (round bottom, Corning). For the other assays, the cells were centrifuged at 500g for 5 minutes at RT, resuspended in PBS and transferred to the 96-well plate followed by centrifugation. In the meantime, fixable viability stain (FVS450) was prepared by dilution in PBS. 100 µl FVS450 was added to each well and incubated for 15 minutes at RT, shielded from light. The staining reaction was stopped by washing in FACS buffer consisting of 10% FBS, 1 mM EDTA, and 0.05% (w/v) sodium azide in PBS (Region Hovedstadens Apotek, Denmark). For phenotypic characterization of the lymphocytes a multicolor panel was applied (see **Table 1**). Each antibody was titrated prior to use and the ideal antibody concentration was determined by Staining Index, analyzed in FlowLogic (Inivai, version 700.1A). Prior to antibody staining, Fc-receptors were blocked with 10 µg/mL human Fc-Block for 10 minutes at RT. The concentration was based on data published by Andersen et al. (34). A mastermix of antibodies was prepared and the stained cells were incubated dark for 30 minutes at RT followed by two washes with FACS buffer. The samples were acquired for 120 seconds on a FACSLyric 3-laser 10-color CE-IVD flow cytometer (BD Bioscience) and analyzed in FlowLogic.

**Table 1:** Detailed list of reagents and concentrations applied for flow cytometry.

Marker	Fluorophore	Clone	Dilution*
CD3	APC-Cy7	SK7 (Leu-4)	1/2x
CD4	PE	OKT4	1/2x
CD8	APC	RPA-T8	1/2x
CD19	PerCP-Cy5.5	HIB19	1/2x
Human BD Fc Block	none	Fc1	10 µg/ml
Proliferation	CFSE		5 µM (1.25-20 µM)
Viability	FVS450		1/2x

*) Relative to the recommended concentrations. All reagents for flow cytometry were obtained from BD Biosciences.

**Figure 1:**
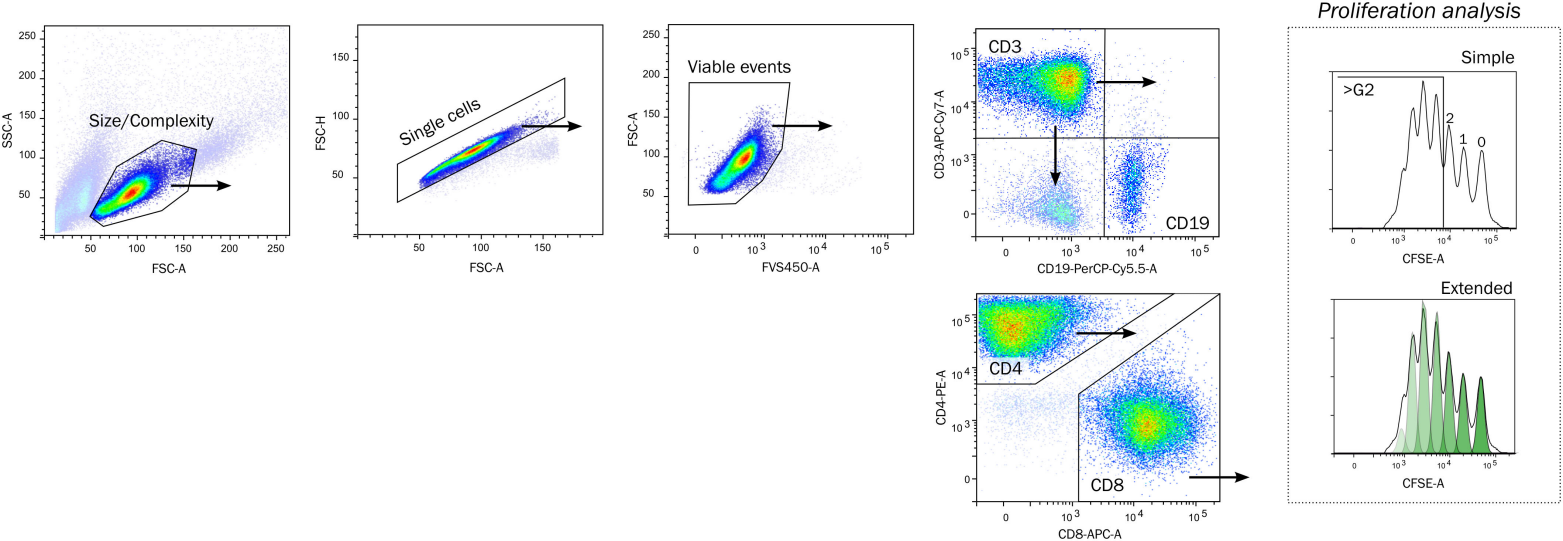
Gating strategy. Lymphocytes were identified by a crude Size and Complexity gate, Single cells by area/height proportionality on forward scatter pulse, and viable as FVS450-negative. The proliferation of lymphocytes was quantified on CD3+ CD19- T cells by a simple or extended proliferation analysis. The simple strategy enumerates cells having divided beyond the second generation, whereas the extended analysis fits a model to calculate metrics (see methods for details). The CD3+ cells were further divided into CD4+ and CD8+ cells. FSC-A/FSC-H, forward scatter area/height, FVS450, fixable viability stain 450; CFSE, Carboxyfluorescein succinimyl ester.

The gating strategy (**Figure 1**) was common for all experimental setups, where the lymphocytes were selected by Size/Complexity, duplets were removed from the analysis, and viable cells were selected for further analysis. The reasoning for size/complexity is presented in **Supplementary Figure 4**. T cells (CD3+, CD19-) were analyzed by a simple gating strategy measuring proliferation of cells beyond the second generation (>G2), or an extended analysis by model fitting. T cells were further divided into CD4 and CD8-positive cells. B cells (CD19+) were neither included for analysis of lymphocyte proliferation for mitogen or MLR assays because the proliferative response was too low to be detected.

The model fitting of proliferating cells enables calculation of six metrics according to Roederer: Fraction diluted/Proliferating (P), Precursor Frequency (PF), Expansion Index (EI), Replication Index (RI), Division Index (DI) and Proliferation Index (PI). A detailed description of each metric is reviewed by Roederer (35). In brief, P denotes the fraction of cells in the entire culture which have divided at least once where PF describes the fraction of responder cells, based on modelling of the original population. EI and RI quantify the fold expansion of entire cell culture and responder cells, respectively. DI is the average number of cell division for all cells in the culture, where PI describes the average number of cell division that responder cells have undergone.

### Statistics

Normality of data was confirmed by Shapiro-Wilk normality test. For pair-wise comparisons, paired or unpaired T tests were performed as appropriate, and for comparisons of more groups, one-way or two-way ANOVAs were applied. Analysis of the effect of dose-response to ASC was performed using a repeated-measures one-way ANOVA with Geisser-Greenhouse correction. For relative half maximal effective concentration (EC_50_), minimum-maximum normalization and non-linear regression by variable slope dose-response curve was fitted. Cryomedia and monocyte-depleted conditions were compared by mixed-effects model. For multiple comparisons, p values were adjusted for false discovery rate (Q=0.05) by the two-stage linear step-up procedure of Benjamini, Krieger and Yekutieli (36), except CFSE-labeling prior to cryopreservation, for which Dunnett’s test was used to obtain 95% confidence intervals. Significance is reported at adjusted levels of *) p < 0.05; **) p < 0.01; and ***) p < 0.001. All statistical analysis was performed in GraphPad Prism 9.3.1 (GraphPad Software, LLC).

## Results

### Ideal mitogen concentrations for testing of ASC

Five mitogens were titrated over a broad range of concentrations. The observed proliferation was restricted to T cells, and no discernible cell divisions were detected on B cells. Thus, the quantification was carried out on T cells (CD3+ CD19-). Instead of focusing solely on the proliferative response elicited, a fixed dose of ASC (at a ratio of 1 ASC for every 5 PBMC; 1:5) was included to determine the effect ASC at the given concentration. To minimize donor variation, a pool of five ASC donors was used. The pool of donors was compared to the included ASC separately to rule out potential inhibiting, additive, or synergistic effects. Two doses of ASC (1:5 and 1:10) were tested, and 1:5 subsequently selected due to the stronger inhibition displayed (**Supplementary Materials Figure 2**).

**Figure 2:**
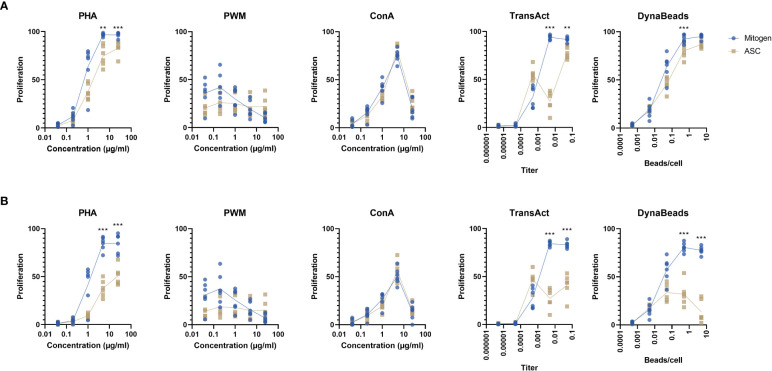
Percentage of proliferating lymphocytes by two different gating strategies. **(A)** The proliferation of all T cells, regardless of generation in response to 5 different mitogens at different concentration in absence or presence of ASC (blue and yellow dots, respectively). **(B)** The >G2 gating strategy was applied to determine T cell proliferation beyond the second generation. Paired t test. Significance levels of **) p < 0.01; and ***) p < 0.001. n=6. PHA, Phytohemagglutinin; PWM, Pokeweed mitogen; ConA, Concanavalin A; ASC, Adipose tissue-derived Stromal Cells.

For the unspecific mitogens, PHA and ConA induced a strong proliferative response, while the pattern was less discernible for PWM which did not exceed 50% at any concentration (**Figure 2A**). The specific TransAct and Dynabeads generated a response closely resembling PHA. Addition of ASC reduced the proliferation in PHA, TransAct, and Dynabeads, but not PWM or ConA. The inhibition was especially evident at a titer of 1:200 of TransAct. By focusing on later generations of proliferation beyond two divisions (**Figure 2B**), the effect of ASC is emphasized, particularly on PHA and Dynabeads.

Based on these finding, we determined the ideal concentrations to be 5 µg/ml PHA, 5 µg/ml ConA, 1:200 TransAct, and 0.5 beads/cell Dynabeads. PWM was dropped from the study as the data did not allow for analysis, i.e., absence of proliferation and distinct CFSE peaks.

### Suppressive effect of ASC on proliferation metrics

**Figure 3:**
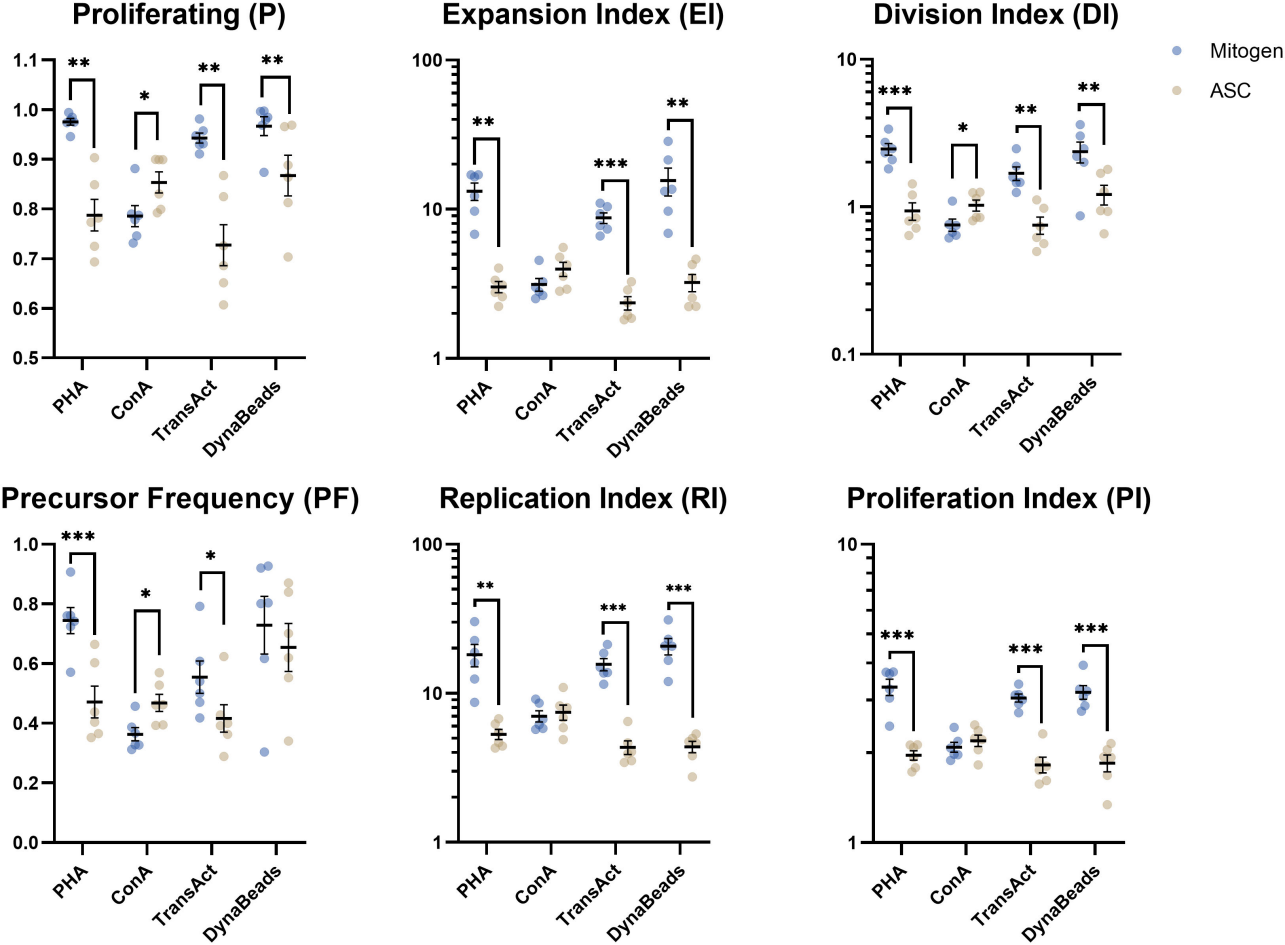
Analysis of 6 proliferative metrics by model fitting of T cells. PBMC were stimulated with four different mitogens at optimum concentration in absence or presence of ASC (blue and yellow dots, respectively) to demonstrate the inhibitory effect on lymphocyte proliferation. Paired t test. Significance levels of *) p < 0.05; **) p < 0.01; ***) p < 0.001. n=6. PBMC, Peripheral Blood Mononuclear Cells; PHA, Phytohemagglutinin; ConA, Concanavalin A; ASC, Adipose tissue-derived Stromal Cells.

Once the concentrations of mitogens had been pinpointed, more elaborate analyses could be performed by curve fitting (**Figure 3**). The inhibition on PHA, TransAct, and Dynabeads was significant on the parameters detailing the entirety of the culture (i.e., P, EI, and DI) and focusing on proliferating cells alike (RI and PI). ASC reduced PF on PHA-induced proliferation only. Interestingly, ASC increased some parameters on ConA-treated PBMC significantly (P, PF, and DI) whereas others remained unchanged (EI, RI, PI). Common trends were shared between CD4 and CD8 T cells subtypes (**Supplementary Materials Figure 5**).

### MLR optimization

**Figure 4:**
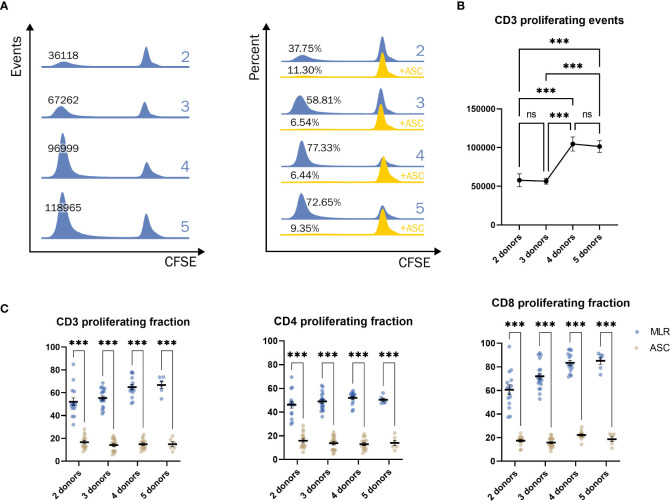
Significant increase in response when combining 4 donors or more. **(A)** Example of the proliferative response of T cells when combining a pool of 2, 3, 4, or 5 donors in events (blue, left) and percentages (blue, right). Addition of ASC greatly reduced the response (yellow, right). **(B)** The number of T cells when combining a pool of 2, 3, 4, or 5 PBMC donors. One-way ANOVA. **(C)** The percentage of proliferating CD3+ (left), CD4+ (middle), and CD8+ (right) cells from different combinations of pooled PBMC donors. Two-way ANOVA. Significance levels of *) p < 0.05; **) p < 0.01; ***) p < 0.001. The number of combinations is described in methods. PBMC, Peripheral Blood Mononuclear Cells; MLR, mixed lymphocyte reaction; CFSE, Carboxyfluorescein succinimidyl ester; ASC, Adipose tissue-derived Stromal Cells; ns, not significant.

In order to find the ideal combination of PBMC donors to induce an allogeneic response, the PBMC were co-cultured in an MLR of 2, 3, 4, or 5 donors. The population of proliferating cells at different late generations does not allow for curve fitting (**Figure 4A**), and therefore, a simple gating strategy was applied. The proliferative response was enhanced by combining multiple PBMC donors, presented by increased number of events (**Figure 4A** left). ASC potently inhibited the proliferative response independently of the number of pooled PBMC (**Figure 4A** right). A significant increase in the proliferative response was observed when combining 4 or more PBMC donors (**Figure 4B**). The fraction of CD3 positive proliferating cells increased with number of donors included, highly driven by the response of CD8 positive T cells, while the fraction of CD4 positive cells was not affected (**Figure 4C**). Regardless of T cell subset, ASC greatly reduced the fraction of proliferating cells.

### Ranking of mitogens

**Figure 5:**
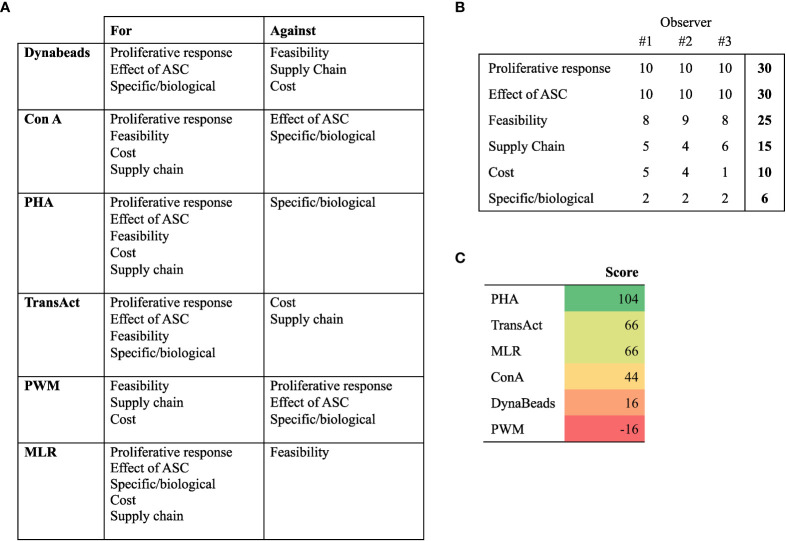
Rankings of mitogens and MLR to determine their suitability for lymphocyte proliferation potency assay. **(A)** Distribution of terms for or against a given mitogen or MLR. **(B)** Weight attributed to terms, ranging from 10 (very important) to 0 (not important). The sum of 3 observers is used to compute the score. **(C)** Score of mitogens derived from subtracting terms against from terms for. PHA, Phytohemagglutinin; ConA, Concanavalin A; ASC, Adipose Tissue-derived Stromal Cells; MLR, Mixed lymphocyte reaction.

Besides experimental read-outs, other considerations are of importance for potency assays. Six relevant terms were identified: 1) Proliferative response: the mitogen should result in a consistent quantifiable proliferative response of lymphocytes; 2) Effect of ASC: for assessment of potency, ASC must have an effect on the proliferative response; 3) Feasibility: to minimize inter-/intra-assay variation and the risk of user error, the assay should be practical and easy to perform; 4) Supply Chain: having multiple vendors or a generic product increases the availability the mitogen; 5) Cost: the price of the mitogen per assay; 6) Specific/biological: for some applications, it may be relevant to mimic specific or biological interactions (**Figure 5**). Three observers ranked the importance of the terms on a scale from 0-10 (least to most). The sum of the three was used to calculate a score, derived from subtracting the sum of terms “against” from the terms “for”.

Based on the ranking, we decided to focus on PHA. It should be noted we consider all tested assays, apart from PWM, valid for development of potency assays.

### ASC dose-response

**Figure 6:**
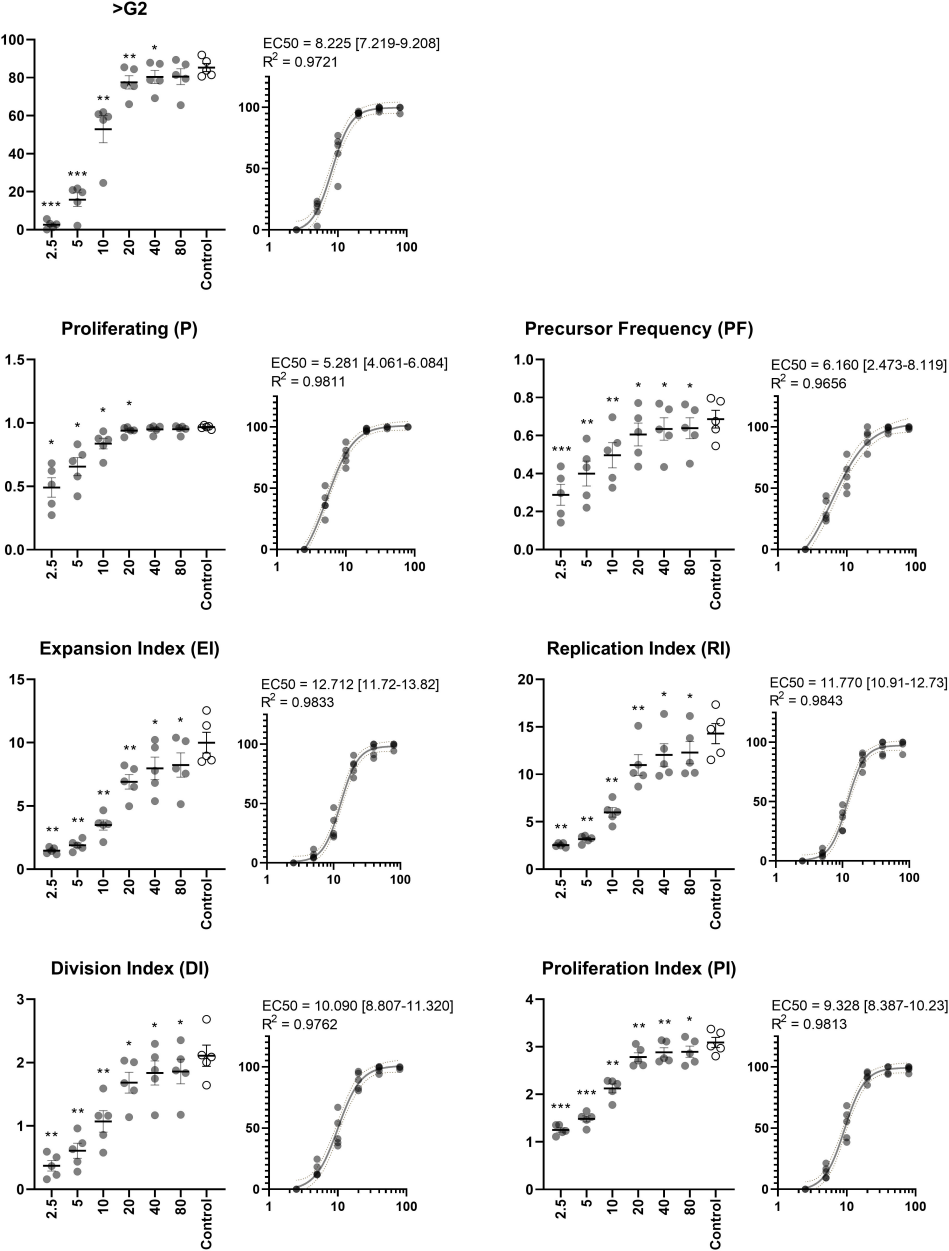
Dose-response of ASC. Percentage of proliferating lymphocytes by simple analysis (>G2) and extended analysis (P, PF, EI, RI, DI, and PI). ASC were included in concentrations (ASC : PBMC ratios) ranging from 1:2.5 to 1:80. EC_50_ was calculated to determine the ratio at which half maximum effect occurs. The PHA-stimulated control does not contain ASC Repeated-measures ANOVA. Significant differences from the PHA-stimulated control are indicated at levels of *) p < 0.05; **) p < 0.01; ***) p < 0.001. EC_50_ is reported with 95% confidence interval in square brackets and goodness of fit (R^2^) inserted above dose-response curves. n=5. PHA, Phytohemagglutinin; EC_50_, half maximal effective concentration; ASC, Adipose tissue-derived Stromal Cells; PBMC, Peripheral Blood Mononuclear Cells.

Rather than assessing a single concentration of ASC, we sought to elucidate how the effect of escalating cell doses affect the proliferative output. 5 independent PBMC donors were stimulated with PHA. As previously described, a pool of ASC was used to minimize variation and produce results reflecting multiple donors. With increasing concentrations of ASC, a marked decrease in proliferation was observed (**Figure 6**). The effect was least evident on P and PF, as many lymphocytes will initiate proliferation in response to PHA regardless of ASC, however, the extent was more convincing on the remaining metrics (>G2, EI, DI, RI, and PI).

In the optimized assay, very uniform tendencies were observed for PBMC donors and experiments. It can be challenging to compare potencies, and we therefore sought to assess EC_50_ as a tool to ease this process. PHA induces a response in a high fraction of lymphocytes and creating conditions which eliminate the response altogether require much higher concentrations of ASC, which is reflected in P and PF (EC_50_ = 5.281 and 6.160, respectively). The actual concentration is likely higher, as we observe no clear evidence of achieving a maximum effect. To gain this insight, even higher concentrations of ASC are required (e.g., 1:1 or even 2:1). However, the remaining metrics displayed the characteristic sigmoidal curve and EC_50_ was well within the range of ratios. The effect on EI and DI reached half at ratios of 12.712 and 10.090, respectively. Slightly higher doses were required to obtain a similar effect when focusing solely on proliferating cells in terms of RI and PI (EC_50_ = 11.770 and 9.328, respectively).

### Ideal cryopreservation medium and monocyte depletion

**Figure 7:**
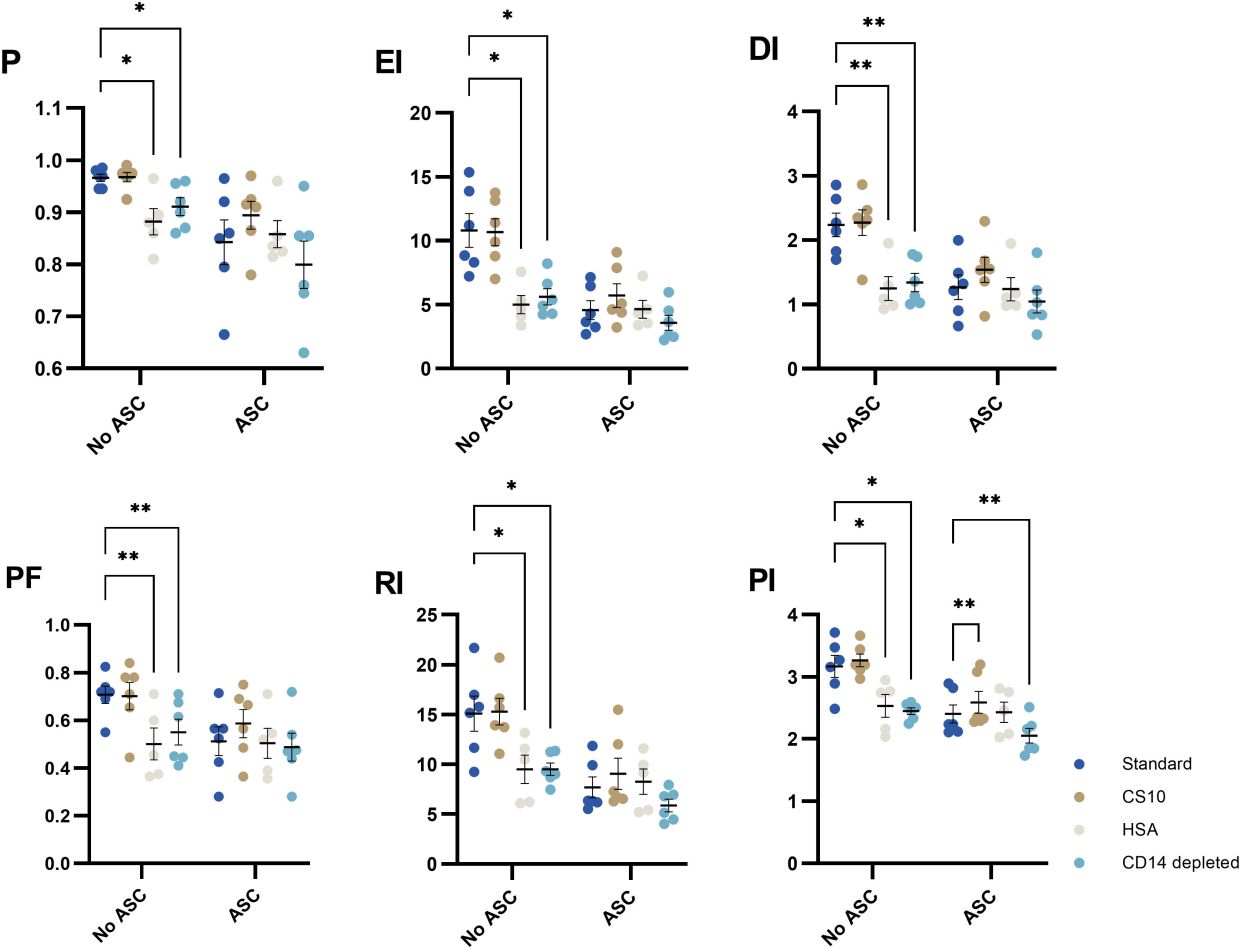
The impact of cryomedia and monocyte-depletion on the proliferative response of PHA-stimulated T cells in absence or presence of ASCs, represented by 6 proliferative metrics (P, EI, DI, PF, RI and PI). PBMC were either cryopreserved in 90% FBS with 10% DMSO (standard), 12.5% HSA in RPMI with 10% DMSO, or CS10. Monocyte-depleted cells were cryopreserved in standard. Mixed-effects model. Significance levels of *) p < 0.05; **) p < 0.01. n= 6 except for HSA (n=5). PHA, Phytohemagglutinin; ASC, Adipose tissue-derived Stromal Cells; PBMC, Peripheral Blood Mononuclear Cells; HSA, Human Serum Albumin; CS10, Cryostor CS10; P, Fraction diluted/Proliferating cells; EI, Expansion Index; DI, Division Index; PF, Precursor Frequency; RI, Replication Index; PI, Proliferation Index.

Cryopreservation in FBS in combination with DMSO is common and has been optimized in-house previously (29). For development of potency assays, GMP-grade reagents are required. Some assays require formulations devoid of xenogeneic components. To test formulations of medium for cryopreservation (cryomedium), we included the comparators CS10 as a ready-to-use GMP-grade reagent and HSA (12.5% HSA, 10% DMSO) as a human derivative. No significant differences were observed between standard FBS/DMSO and CS10 (**Figure 7**), but proliferation was significantly reduced in the HSA cryomedium.

Seeing that the PBMC are derived from blood donations, we sought to separate monocytes from lymphocytes in an attempt to preserve these for other uses. Interestingly, depletion of monocytes significantly impairs the proliferative response of lymphocytes (**Figure 7**).

### CFSE-labelling prior to cryopreservation

**Figure 8:**
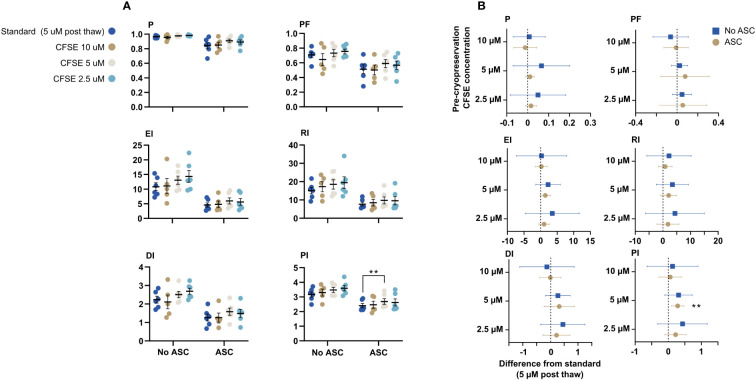
CFSE-labelling prior to cryopreservation. PBMC were labelled with CFSE at 3 different concentrations: 2.5, 5, and 10 µM prior to cryopreservation or with 5 µM after thawing (standard). **(A)** The impact on the six proliferative metrics (P, EI, DI, PF, RI and PI) for T cells when the PBMC were labelled prior to cryopreservation compared to standard. The cells were stimulated with PHA in presence or absence of ASC. **(B)** Mean difference from standard (dotted line) with confidence intervals. Mixed-effects model with Dunnett’s test for multiple comparisons and 95% confidence interval. n= 6, except for 10 µM CFSE (n=5). CFSE, Carboxyfluorescein succinimidyl ester; ASC, Adipose tissue-derived stromal cells; PBMC, Peripheral Blood Mononuclear Cells; PHA, Phytohemagglutinin; P, Fraction diluted/Proliferating cells; EI, Expansion Index; DI, Division Index; PF, Precursor Frequency; RI, Replication Index; PI, Proliferation Index.

We tested the option to label the PBMC with CFSE prior to cryopreservation to increase feasibility. Labelling was performed with three concentrations: 2.5, 5, and 10 µM. For all metrics, there were no difference of the proliferative response compared to PBMC labelled with 5 µM CFSE post thawing (standard conditions). The suppressive effect of ASC was significantly different for PI between standard and 5 µM CFSE, but not for the other metrics (**Figures 8A, B**). In general, the proliferative response of PBMC labelled with 2.5 or 5 µM was slightly higher compared to standard, however, not statistically significant, where 10 µM CFSE prior to cryopreservation was most similar to 5 µM post thawing (**Figure 8B**).

## Discussion

In this study, we demonstrated that lymphocyte proliferation is affected by the mitogen and its concentration, but more importantly, that the effect of ASC varies. We showed that ConA induces a considerable response at a concentration of 5 µg/ml, but the suppressive effect of ASC was absent or tended to promote the proliferative response in contrast to reported findings (37). Furthermore, we showed that PWM was able to induce proliferation of lymphocytes at low concentrations, however, a very modest response compared to the other investigated stimuli. PWM has been used for decades and the extent of lymphocyte proliferation reported lower than PHA (38). Although it has been suggested that PWM induces proliferation of B-cells (26, 39), we did not observe an increased B cell population in our cell culture (data not shown), and hence not a suppression by ASC. We cannot rule out that ConA and PWM can be applied in other assays, but it requires further optimization in our hands. Our results suggest that the response elicited by PHA, TransAct, and Dynabeads, and the suppressive effect of ASC on these mitogens, is more promising for immunomodulatory potency assays.

Prior to finding the ideal mitogen concentration, the suppressive effect ASC was tested at different ratios: 1:5 and 1:10 (ASC : PBMC). Previously, we have shown that a ratio at 1:10 is sufficient to inhibit lymphocyte proliferation (7, 29). However, in this study the suppressive effect of ASC was more evident at a ratio 1:5 (**Supplementary Materials Figure 2**). Thus, this ratio was applied to ensure near maximal inhibitory effect of the ASC.

The use of CFSE enables quantification of proliferating generations by flow cytometry, which allows for gating on the later generations, e.g., cells beyond the second generation (>G2). Using this gating strategy, we have previously demonstrated that later generations are more affected by the suppressive effect induced by ASC (7), which is especially true for PHA and Dynabeads.

In contrast to the dependence of concentrations of mitogens for optimal quantification of the suppressive potential of ASC, MLR is dependent on number of PBMC donors, but displays a profound inhibition by ASC in all instances. We tested combinations of 2-5 donors and found a significant increase in proliferation by including 4 donors, but the response was not augmented by including an additional donor. This is in agreement with Mangi et al, who demonstrated that no more than 4 individual donors are needed to obtain maximum response of lymphocytes in an MLR (28). The combination of multiple PBMC donors in a MLR deviates from the conventional methodology of a one-way MLR, where responder cells (from one donor) are allogeneic stimulated by another irradiated or chemically treated donor (non-proliferative stimulator cells) to investigate a specific response (40, 41). However, the aim of this study was to obtain a maximal proliferative and reproducible response induced by allogenic mismatches of a sufficient number of PBMC donors to assess the suppressive effect of ASC. Ketterl et al. monitored the response of 10 pooled PBMC donors on a 3 to 7 days’ time course and found that 7 days resulted in the highest proliferative response of lymphocytes (17), where we settled on 8 days (**Supplementary Materials Figure 3**). We demonstrated that the suppressive effect of ASC on the lymphocyte proliferation of CD3, CD4, and CD8 positive T cells were not affected by the number of pooled PBMC. Nicotra et al. use the MLR model developed by Ketterl et al., with minor changes in the methods, and validated the model according to guidelines of ICH (Q2) R1 which is required for implementing a potency assay (22). Altogether, these data indicate that MLR can be a suitable potency assay for MSC.

By including multiple donors, it is possible to maximize proliferation, yet some advantages pertain to the use of single donors. Besides the practical considerations of easier processing and less consequences of a donor failing when multiple PBMC donors are assayed in parallel, it is possible to study specific ASC-PBMC donor interactions and address aspects such as human leukocyte antigen matching.To find the most suitable immune potency assay for our cell-based product, we listed 6 aspects (Proliferative response, Effect of ASC, Feasibility, Supply Chain, Cost, and Specific/biological) and ranked the 5 different mitogens and MLR (**Figure 5**). ConA and PWM was excluded as they did not produce an adequate proliferative response of lymphocytes or an effect of ASC, thus leaving PHA, TransAct, Dynabeads, and MLR as candidates. Despite the promising prospect of MLR as an immune potency assay, we assessed that the feasibility could be compromised when simultaneously purifying, labelling, and cryopreserving PBMC from 4 individual donors. Additionally, the flow cytometric data generated from MLR does not allow for extended analysis by curve fitting, which provides additional information. The mitogens, on the other hand, provided very distinguishable peaks suited for curve fitting. TransAct was a promising candidate especially because it is GMP compliant, but the supply chain is limited to one manufacturer. If the product is out of stock or discontinued, the quality control is stalled and consequently the batch not released for clinical use. Dynabeads is also limited to one manufacturer and require more handling. Any additional handling reduces feasibility while increasing the risk of introducing operator errors. Thus, we decided to apply PHA in our potency assay. Krampera et al., 2013 argue not to use PHA and unfractionated PBMC for potency assays due to high variability, mainly because the number of monocytes varies between donors which may affect the results and secondly that PHA is an unspecific mitogen (42). However, we overcome this issue by making a cell bank of “ready-to-use” CFSE-labelled PBMC, making it possible to repeatedly use identical immune cells to test the quality of multiple ASC batches and including multiple PBMC donors in parallel. Our data suggest that the effect of ASC on PHA-stimulated PBMC closely resembles more specific means of activation, i.e., CD3/CD28 stimulation. It is important to emphasize that the ranking of the assay should be performed on a laboratory-level, and the decision of using PHA resemble the opinion of the authors. MLR, TransAct, and Dynabeads are valid candidates for the development of potency assays.

In this study, we focused on creating the assay with least variability between PBMC donors. To minimize variation from ASC donors and rule out results that were unique to a single donor, we pooled an equal number of viable ASC from 5 different donors. We demonstrated that there was no difference in the mean inhibitory effect of using single donors compared to pooled ASC (**Supplementary Figure 2**). This in an agreement with Ketterl et al, which showed that when pooling 5 individual donors, the inhibitory effect was a mean of across individual donors (17). They did, however, observe a significant difference between of the inhibitory effect of individual MSC donors. Pooled MSC does not result in a synergic effect but reduces the variance observed across donors. By using a pool of ASC donors for designing an assay, a mean effect is achieved and the risk of coincidentally drawing conclusions from “good” or “bad” donor eliminated. The alternative to this is inclusion of multiple ASC donors, which increases the number of analyses, reagents, and ultimately costs substantially.

The proliferative response of lymphocytes stimulated with PHA is suppressed in a dose-dependent manner by ASC (**Figure 6**). This is in an agreement with other studies across different proliferation assays and MSC sources (7, 17, 22, 37), suggesting that high concentrations of MSC are able to suppress proliferation independent of exact methodology of the assay. Including several concentrations of MSC to generate data on dose-response relationships are crucial because it enables quantification of potency at an extent, which a single dose does not, and ultimately discriminate potencies of MSC batches. Different methodologies can be applied to quantify potency. We used EC_50_ calculations to determine the ratio at which half the inhibitory effect was observed and achieved very reproducible results between PBMC donors. A similar methodology will be pivotal for potency assay validation and examining the potency of future ASC batches.

Cryopreservation was performed in 3 formulations, of which 2 were xeno-free: HSA with DMSO, CS10, besides standard (FBS with DMSO). We showed that the proliferation of PBMC cryopreserved in CS10 was comparable to standard, but a significant difference between standard and HSA. In contrast, Disis et al. reported that the stimulation index of fresh PBMC stimulated with PHA was similar to HSA-cryopreserved PBMC, (but lower when stimulated with tetanus toxoid), while standard cryomedium did not impair proliferation (31). Other studies have shown that the viability of PBMC cryopreserved in HSA is comparable to standard (30, 32); however, one of the studies (Schulz et al) indicate that the functionality of T cells was slightly impacted when cryopreserved in HSA compared to standard. The presented results indicate that HSA can be used as cryomedium with caution. As mentioned, we showed promising results of using CS10 as cryomedium for PBMC. This was in agreement with Haastrup et al, which showed high viability and recovery after thawing of purified T cells (> 90%) from PBMC, and an unperturbed proliferative response, when cryopreserved in CS10 (43). Additionally, CS10 is manufactured in compliance with GMP making the cryomedium tempting for a potency assay and a promising xenofree alternative.

Furthermore, we investigated the possibility of using monocyte-depleted PBMC for the potency assay, making the CD14+ cells available for other assays, such as studies on monocytes, macrophages, or dendritic cells (8, 44, 45) and thus utilize the full potential of a buffy coat. However, we observed an impaired response (on all metrics) of monocyte-depleted PBMC, demonstrating that monocytes are important for achieving maximum response when stimulated with PHA. This is likely in part through secretion of soluble mediators from monocytes that enhance lymphocyte proliferation (24, 46).

To raise the feasibility, CFSE-labelling prior to cryopreservation was tested at 3 different concentrations (2.5, 5, or 10 µM CFSE). We found no statistically significant difference across the three concentrations compared to standard (5 µM post thawing), which demonstrates the robustness of the method; similar results can be generated regardless of minor deviations in concentration of CFSE. 10 µM CFSE yielded results most similar to the standard methodology. In future assays, PBMC will be stained with 10 µM CFSE prior to cryopreservation in CS10.

Collectively, the present study prompts a design of an immune potency assay of lymphocyte proliferation. The potency assay will consist of a cell bank of PBMC labelled with 10 µM CFSE cryopreserved in CS10, which enables quality and potency tests of ASC batches. Proliferation is induced by 5 µg/ml PHA for 5 days in the presence of escalating doses of ASC, followed by data acquisition by flow cytometry. As part of implementation, the assay will be validated according to legislation by European Commission (20) prior to inclusion in the quality control system. A single potency assay cannot suffice in quantifying the function of MSC (21), as the therapeutic potential span multiple diverse properties (e.g., immunomodulatory, anti-apoptotic, angiogenic, wound-healing, etc.), and a potency assay must reflect the mode of action observed in clinical indications. In presenting our approach to establish an optimized lymphocyte proliferation assay in detail, we hope to aid others in developing *in vitro* models suitable for potency assays.

## Data availability statement

The raw data supporting the conclusions of this article will be made available by the authors, without undue reservation.

## Ethics statement

The studies involving human participants were reviewed and approved by Region Hovedstaden Regional Research Ethics Committee. The patients/participants provided their written informed consent to participate in this study.

## Author contributions

SH: Conceptualization, methodology, validation, formal analysis, investigation, writing - original draft, visualization. LH: Validation, resources, writing - review & editing. JK: Writing - review & editing, supervision, funding acquisition. AE: Writing - review & editing, supervision, funding acquisition. BF: Writing - review & editing, project administration. MJ: Conceptualization, methodology, formal analysis, investigation, writing - original draft, visualization. All authors contributed to the article and approved the submitted version.
