# Supplementary material for: Optimizing an immunomodulatory potency assay for Mesenchymal Stromal Cell
Source: Front Immunol. 2022 Dec 12;13:1085312. doi: 10.3389/fimmu.2022.1085312 (PMC9791065; doi:10.3389/fimmu.2022.1085312)
Supplement: Supplementary file 1 [file DataSheet_1.docx]

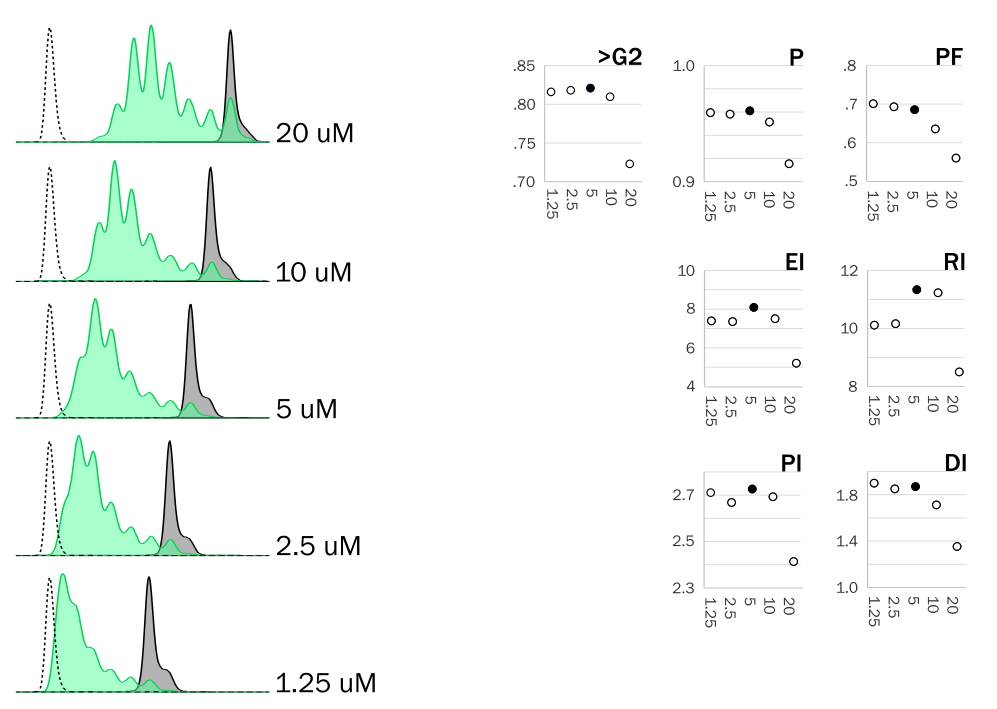


**Figure 1:** Titration of CFSE. Representative data from one donor of four PBMC donors. Staining displayed distinct peaks for quantification. The proliferative response was robust, and only at the highest concentration (20 µM), a reduction was observed. At a concentration of 5 µM, the histograms were ideal for manual analysis (>G2) and the proliferative potential was not affected compared to lower doses. Increasing the PMT voltage allows for even better discrimination between peaks, but for comparison with higher concentrations, the voltage was kept constant. CFSE: Carboxyflourescein succinimidyl ester; PMT: Photomultiplier tube; P: Fraction diluted/Proliferating cells; EI: Expansion Index; DI: Division Index; PF: Precursor Frequency; RI: Replication Index; PI: Proliferation Index.

| 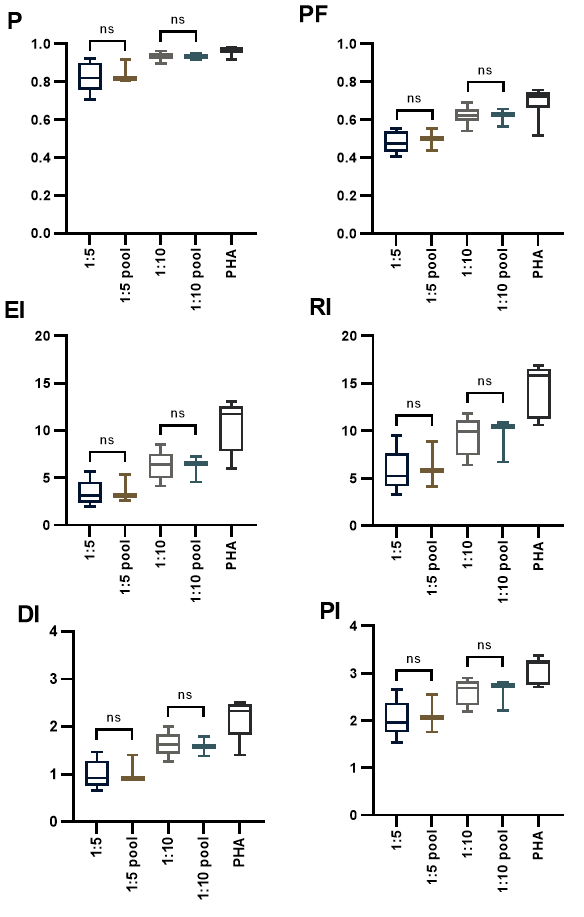 | 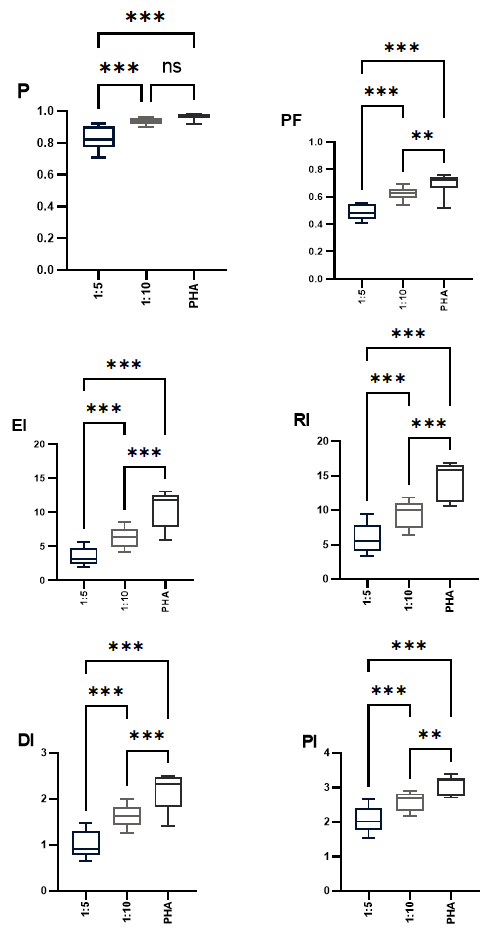 |
| --- | --- |

**Figure 2:** Effect of pooled versus single donor ASC. Six metrics for lymphocyte proliferation of three PBMC donors, which were co-cultured in absence and presence of five single ASC donors or a pool of the same ASC donors at different ratios: 1:5 or 1:10 (ASC:PBMC). The PBMC were seeded one day after the ASC and the cells were cultured for five days in complete medium supplemented with 5µg/ml PHA. There was no statistical difference on suppressive effect of ASC on the lymphocyte proliferation between the mean of single ASC donor and pooled donors. However, the inhibitory effect of ASC at a ratio of 1:5 was significantly higher compared to 1:10 . Therefore, it was concluded to use pooled ASC from five donors at a ratio at 1:5 . One-way anova, FDR adjusted significance. Significance levels of *) p < 0.05; **) p < 0.01; ***) p < 0.001. n=3 (PBMC) in one experimental setup. ASC: Adipose tissue-derived Stromal Cells; PBMC: Peripheral Blood Mononuclear Cells; PHA: Phytohaemagglutinin; P: Fraction diluted/Proliferating cells; EI: Expansion Index; DI: Division Index; PF: Precursor Frequency; RI: Replication Index; PI: Proliferation Index; FDR: False discovery rate


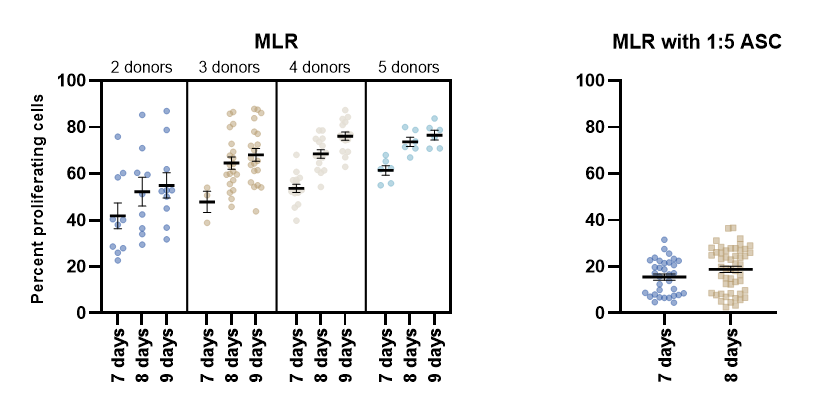


**Figure 3**: Time course of mixed lymphocyte reactions. Six PBMC donors were seeded in a pool of 2, 3, 4 or 5 PBMC donors and were co-cultured in absence and presence of five pooled ASC donors at a ratio of 1:5 (ASC:PBMC) for 7, 8, or 9 days, which were seeded one day prior to the PBMC. The percentage of proliferating PBMC increased with increasing culture time for all numbers of pooled donors. Little to no difference of the proliferative response was observed between day 8 and 9, which could indicate that external or other biological factors affect the proliferative response at day 9 (left figure). The percentage of proliferating lymphocytes for all numbers of pooled PBMC donors co-cultured with ASC for day 7 and 8 (right figure). ASC were not included at day 9 due to scarcity. The suppressive effect of ASC on the lymphocyte proliferation was quite similar at day 7 and 8. Therefore, it was concluded to culture the cells for 8 days. MLR: Mixed lymphocyte reaction. The number of combinations is described in methods. ASC: Adipose tissue-derived Stromal Cells; PBMC: Peripheral Blood Mononuclear Cells.


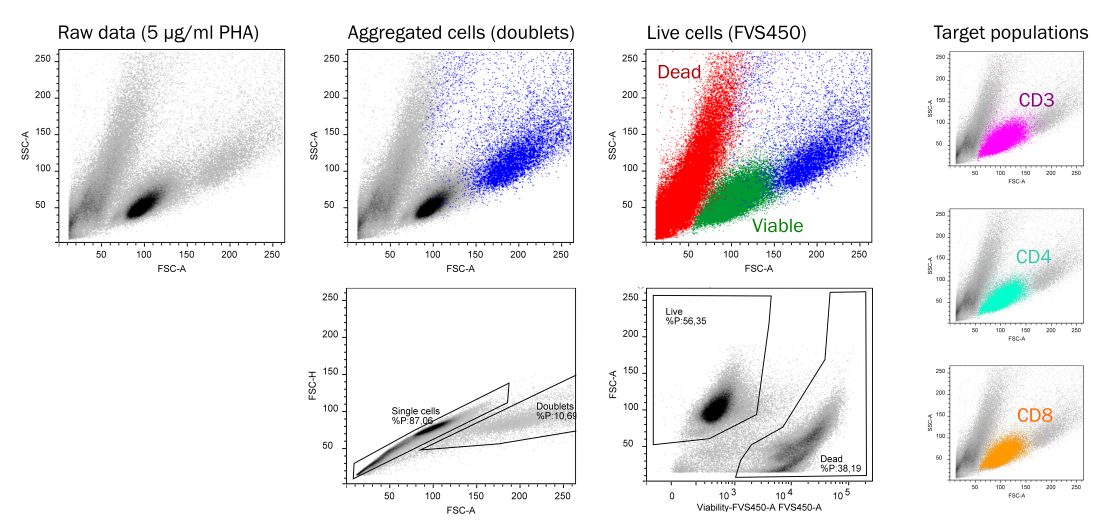


**Figure 4:** Reasoning for Size/Complexity gating. Raw scatterplot showing size (forward scatter, FSC-A) and complexity (side scatter, SSC-A). Cell aggregates (often doublets) are removed by a Single cell gate. By backgating, the doublets can be identified as a minor population in the original plot. Next, the Live cells are identified by FVS450. In the original scatterplot, the live and dead populations are clearly discernable. As target populations (e.g. CD3, CD4, CD8) are all based on single, viable cells, these are naturally confined to the same original population. By using a crude size/complexity gate to identify the population of interest, the risk of artefacts is reduced (e.g., cell debris or freak events).

| 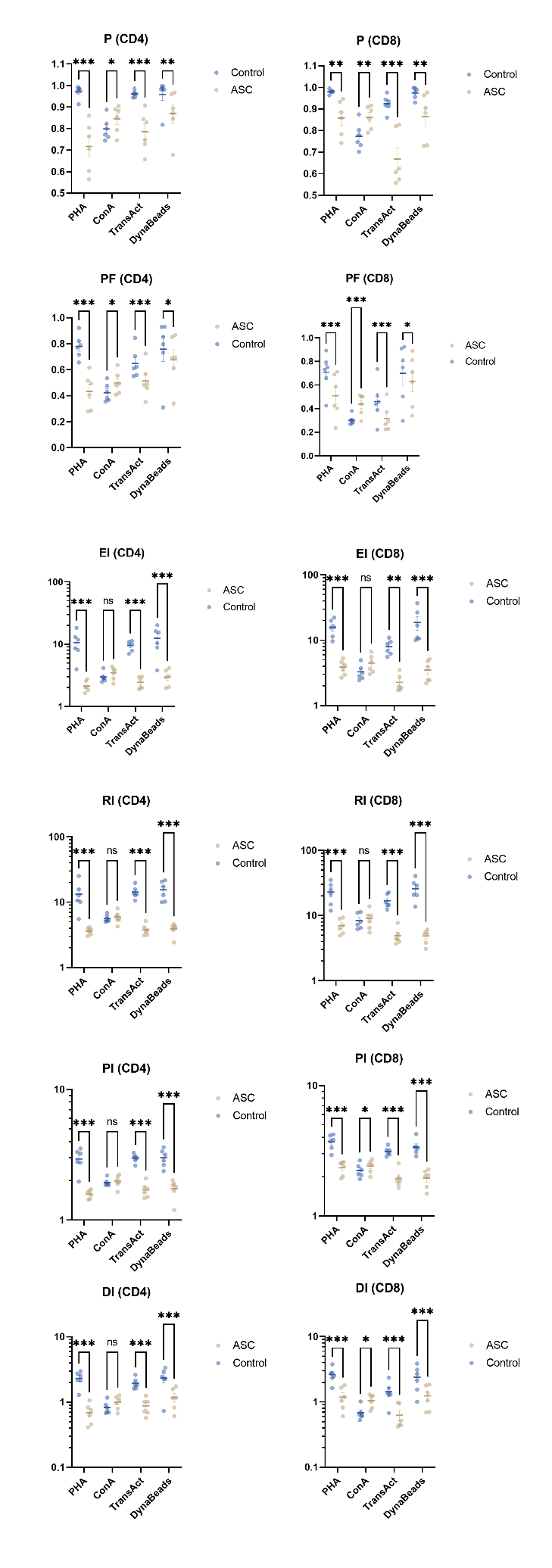 | **Figure 5:** Six metrics (P, PF EI, RI, PI and DI) of the proliferative response for CD4+ and CD8+ T cells, when the PBMC were stimulated with 5µg/ml PHA or ConA, 1:200 titer TransAct or 0.5 bead/ml (Dynabeads) in absence (blue dots) and presence (yellow dots) of ASC (a pool of five donors) at a ratio 1:5 (ASC:PBMC) for five days. Two-way repeated measures ANOVA with FDR (Q=0.05) for multiple comparisons. Significance levels of *) p < 0.05; **) p < 0.01; ***) p < 0.001. P: Fraction diluted/Proliferating cells; EI: Expansion Index; DI: Division Index; PF: Precursor Frequency; RI: Replication Index; PI: Proliferation Index; FDR: False discovery rate. n= 6 (PBMC) |
| --- | --- |
